# Do Statins Affect Viral Infections Encountered by International Travelers?

**DOI:** 10.3390/tropicalmed10030073

**Published:** 2025-03-11

**Authors:** Chinmay T. Jani, Christian Mouchati, Nour Abdallah, Ruchi Jani, Loukas Kakoullis, Lin H. Chen

**Affiliations:** 1 Division of Medical Oncology, Sylvester Comprehensive Cancer Center, University of Miami, Miami, FL 33136, USA; ctjani1494@gmail.com; 2 Division of Neurology, University of Connecticut, Farmington, CT 06030, USA; mouchati@uchc.edu; 3 Department of Medicine, University of Connecticut, Farmington, CT 06030, USA; abdallah@uchc.edu; 4 Department of Medicine, Smt NHL Municipal Medical College, Ahmedabad 380006, Gujarat, India; ruchi.jani27@gmail.com; 5 Department of Medicine, Mount Auburn Hospital, Cambridge, MA 02138, USA; loukas.kakoullis@mah.harvard.edu; 6 Harvard Medical School, Boston, MA 02115, USA

**Keywords:** immunomodulation, statin, COVID-19, influenza, flavivirus, Ebola, hepatitis, norovirus

## Abstract

Statins are among the most frequently prescribed medications. In addition to their well-established effectiveness in lowering total cholesterol, LDL, and triglycerides, statins have been described to have immunomodulatory and anti-inflammatory properties and have been associated with improved endothelial functions. Given the common use of statins, we sought to evaluate the effect of statins on some viral infections encountered by residents in tropical areas or by international travelers. A literature search was performed in PubMED/MEDLINE focusing on keywords that included statins and the viruses of interest, including SARS-CoV-2, influenza, yellow fever, dengue, Zika, tick-borne encephalitis, hemorrhagic fever viruses, hepatitis A, norovirus, hepatitis B, hepatitis C, measles, and herpesviruses; findings were synthesized for each virus into a summary. The effects of statins on viral infections vary depending on the specific virus. While some studies indicate potential benefits in chronic HBV and HCV infections, evidence regarding SARS-CoV-2 and influenza remains inconclusive due to mixed findings from observational studies and randomized controlled trials. The role of statins in other viral infections is largely unexplored, with preclinical data available for only a few viruses. Given the conflicting evidence, further prospective studies and randomized controlled trials are warranted to elucidate statins’ role in viral infections, particularly in modulating inflammation, endothelial dysfunction, and immune responses. Future research should aim to define the optimal patient populations, target viruses, statin types, and treatment durations that may confer benefits in specific viral infections.

## 1. Introduction

Over 200 million people worldwide are estimated to be on statin therapy [1]. In the United States (US), statins are among the most commonly prescribed drugs [2,3]. They are primarily known to function in the treatment of dyslipidemia by inhibiting hydroxymethylglutaryl-CoA (HMG-CoA) reductase, a rate-limiting step in cholesterol biosynthesis. As a result, hepatic cholesterol synthesis decreases, leading to higher microsomal HMG-CoA reductase production and higher cell surface low-density lipoprotein cholesterol (LDL) receptor expression, enhancing the clearance of circulating levels from the bloodstream [4]. Indeed, statins have been proven to lower LDL and triglyceride levels by 20–50% and 10–20%, respectively, and potentially increase serum high-density lipoprotein cholesterol levels by 5–10% [5,6]. While statins are effective and safe medications, they can cause side effects ranging from mild myalgia to debilitating rhabdomyolysis [7] and more serious side effects including new-onset type 2 diabetes mellitus, neurological and neurocognitive effects, hepatotoxicity, and renal toxicity [8].

Given their immunomodulatory action, statins have been evaluated as potential therapeutic agents in viral infections [9,10]. Research indicates that statins modulate immune function by interfering with the mevalonate pathway, which plays a crucial role in antigen presentation. This interference leads to a decline in MHC class II, CD80, and CD86 expression, thereby weakening T-cell activation and proliferation. The impact is particularly evident in B cells, where statins significantly suppress their proliferation and adhesion, ultimately compromising their ability to present antigens effectively [11]. In addition, statins reduce the expression of T-cell surface receptors for viruses such as C-C chemokine receptor type 5 (CCR5) and alter the secretion of proteins and cytokines [12].

Furthermore, by inhibiting the intracellular production of cholesterol, statins deprive the viruses of an important compound that contributes to sustaining their infectious cycles [13]. Cholesterol plays a crucial role in the infectivity of enveloped viruses by preserving the integrity of the viral envelope, facilitating membrane fusion, and supporting viral assembly [14,15]. It also contributes to replication by organizing lipid rafts, which serve as functional platforms for viruses such as Zika, dengue, and influenza A [16,17]. Additionally, cholesterol modulates signaling pathways that enhance viral entry and propagation [18,19]. Statins, by inhibiting cholesterol synthesis, also reduce the prenylation of key proteins like Rho and Ras GTPases, which are critical for membrane localization and intracellular signaling. This disruption interferes with viral processes, including entry, replication, and spread, by altering host pathways that viruses exploit, such as cytoskeletal remodeling, lipid raft organization, and immune evasion. For instance, HCV depends on prenylated host proteins for RNA replication, influenza A virus utilizes Rho GTPases for entry and budding, and HIV-1 requires prenylation for proper Gag protein trafficking and maturation [13,20]. Given cholesterol’s extensive role in viral life cycles, targeting its biosynthesis could offer a promising avenue for antiviral strategies.

The SARS-CoV-2 pandemic led to exploration of the effect of statins on COVID-19. Along with that interest, we conducted PubMED/MEDLINE searches to review the impact of statins on the course and outcomes of some prototypes of viral infections that are considered travel-related risks. We focused particularly on the vaccine-preventable viruses with the highest monthly incidence rate in travelers, namely COVID-19, influenza, dengue, and yellow fever [21]. We have also categorized the evidence of these studies based on Eccles et al. [22].

## 2. Effect of Statins on Different Viruses

### 2.1. Respiratory Viruses: COVID-19

The effect of statins on the outcomes of COVID-19 infection has been extensively reviewed in the literature but with varied conclusions. Early international retrospective observations suggested a beneficial effect of statin use before and during hospitalization on the clinical outcomes of COVID-19, notably critical care admission and mortality [23,24,25,26,27,28,29,30,31,32,33,34,35,36,37,38,39,40,41,42,43,44,45,46,47,48,49,50] (Table 1). Similarly, a Mendelian randomization study demonstrated that higher expressions of HMG-CoA reductase (HMGCR) and HMGCR-mediated LDL cholesterol were associated with a higher risk of COVID-19 hospitalization (odds ratio—OR 1.38, 95% CI 1.06–1.81 and OR 1.32, 95% CI 1.00–1.74, respectively) [51]. In addition, continuous statin use was associated with lower in-hospital mortality compared to no statin use and discontinuation of statins [52]. A Canadian prospective cohort study found no statistically significant impact of statins on COVID-19 incidence and outcomes in patients younger than 75 years; however, patients older than 75 years using statins had increased hospitalizations but lower 30-day all-cause mortality [53]. A prospective study in Italy showed that statin use did not affect critical care admission and mortality but significantly lowered the risk of developing acute kidney injury (OR 0.47, 95% CI 0.23–0.95; *p* = 0.036) and C-reactive protein (CRP) levels (*p* = 0.048) [54].

On the other hand, Wander et al. concluded that statins do not affect 30-day COVID-19 outcomes in over 4 million US veterans [55,56]. Similarly, studies in Iran and Denmark failed to demonstrate a statistically significant association between statin use and COVID-19 disease severity and mortality [56,57,58]. The French CORONADO multi-center study of diabetic patients hospitalized for COVID-19 concluded that statin use was associated with a higher risk of death (OR 1.42, 95% CI 1.00–2.02) [59]. A multi-center observational study in Italy also noted an association of statin use with more severe disease (OR 1.7, 95% CI 1.067–2.71; *p* = 0.026) but not mortality [60].

Furthermore, several meta-analyses of cohort studies were published with conflicting results [61]. Multiple meta-analyses found improvement in COVID-19 outcomes in patients taking statins [62,63,64,65,66,67,68,69,70,71,72,73,74,75,76]. On the other hand, a meta-analysis of more than 11 million patients by Harianto et al. showed that statins did not improve COVID-19 outcomes (OR 1.08, 95% CI 0.86–1.35; *p* = 0.50) [77]. The inconsistent results might be confounded by other variables such as age, sex, and comorbidities, notably cardiovascular, genetic, and environmental factors, as well as polypharmacy [61,78]. Another variable to take into consideration is the type of statin studied, as Rossi et al. concluded that simvastatin and atorvastatin reduced mortality in COVID-19 patients, unlike pravastatin and rosuvastatin, which did not [79]. Similarly, Sperry et al. demonstrated that simvastatin was a potent direct inhibitor of SARS-CoV-2 infected cells in vitro, unlike most other statins, which were less effective [80] (Table 2).

Several randomized control trials (RCTs) have investigated the role of statins in COVID-19 outcomes with mixed results. A total of seventeen RCTs evaluating statin therapy in COVID-19 patients have been registered, most of which focus on hospitalized patients, with only one trial addressing post-discharge management. These trials assess both moderate-intensity statin therapy (simvastatin 40–80 mg, atorvastatin 20 mg, or rosuvastatin 5 mg daily) and high-intensity statin therapy (atorvastatin 40–80 mg or rosuvastatin 40 mg daily). The key findings from the completed RCTs are summarized below [81]. The RESIST trial investigated the use of aspirin, atorvastatin, or both in 900 hospitalized COVID-19 patients. The study found no significant reduction in clinical deterioration across the groups (HR for atorvastatin 0.98, 95% CI 0.34–2.79; *p* = 0.97) or in mortality (HR 1.00, 95% CI 0.36–2.77; *p* = 0.99). Notably, atorvastatin did not impact inflammatory markers such as CRP or IL-6 [82]. Likewise, the INSPIRATION-S trial randomized 587 ICU patients to receive either atorvastatin 20 mg or a placebo. The primary composite outcome (venous or arterial thrombosis, ECMO requirement, or all-cause mortality) was not significantly different between groups (OR 0.84, 95% CI 0.58–1.21; *p* = 0.35). However, in a subgroup analysis of patients hospitalized within 7 days of symptom onset, atorvastatin showed a potential benefit in reducing adverse outcomes (OR 0.60, 95% CI 0.37–0.99; *p* = 0.05) [83]. An RCT in Iran involving 156 patients with severe COVID-19 unexpectedly found that atorvastatin worsened clinical outcomes, leading to prolonged hospitalization (median: 7 vs. 4 days; *p* = 0.001) and higher ICU admission rates (18.4% vs. 1.3%; *p* = 0.001). Mortality rates were numerically higher in the atorvastatin group (6.6% vs. 2.6%), though the difference was not statistically significant (*p* = 0.27) [84]. A meta-analysis of five RCTs (*n* = 2,390,730) evaluating statins in COVID-19 did not demonstrate a mortality benefit (OR 0.66, 95% CI 0.51–0.85). However, the heterogeneity among studies was high (I^2^ = 69%) [85]. Another meta-analysis by Xavier et al., which analyzed four RCTs involving 1231 patients, found no significant benefit of statin therapy in COVID-19 outcomes. The study confirmed that statin use did not significantly reduce all-cause mortality (OR = 0.96, 95% CI 0.61–1.51; I^2^ = 13%, *p* = 0.86). Additionally, there was no difference in hospital length of stay between statin-treated and control groups (MD = 0.21, 95% CI −1.74 to 2.16; I^2^ = 92%, *p* = 0.8), indicating that statins did not shorten the duration of hospitalization. The analysis further demonstrated that statin therapy had no impact on ICU admission rates (OR = 3.31, 95% CI 0.13–87.1; I^2^ = 84%, *p* = 0.47) along with no reduction in the need for mechanical ventilation (OR = 1.03, 95% CI 0.36–2.94; I^2^ = 0%, *p* = 0.95) [86].

### 2.2. Respiratory Viruses: Influenza

Similar to COVID-19, the effect of statin on influenza outcomes has been varied. In murine models, the combination of statins and caffeine decreased lung damage and viral replication as effectively as oseltamivir and ribavirin, notably when administered preventatively [87]. However, Radigan et al. demonstrated that statins did not affect the clearance of the influenza A virus from the lung and did not decrease the severity of the induced lung injury or related mortality [88]. In mice infected with influenza, simvastatin did not decrease the morbidity, mortality, or viral titers, and its addition to oseltamivir was no more efficient than oseltamivir alone [89]. In another study, simvastatin was associated with lower survival rates and increased body mass loss compared to virus-infected control mice [90]. Peng et al. used a human epithelial lung cell line to demonstrate that fluvastatin had an anti-inflammatory activity that minimally inhibited influenza virus infection [91].

**Table 1:** Studies investigating the role of statins in COVID-19.

Pathogen	Year	Title of Study	Type of Study	Type of Statin	Key Findings Pertaining to Statins	Category of Evidence
SARS-CoV-2	2022	Effects of statins on clinical outcomes in hospitalized patients with COVID-19 [85]	Meta-analysis of randomized controlled trials	Multiple statins	There was no evidence of clinical benefits of statin use.	Ia
	2023	Effects of statin therapy in hospitalized adult COVID-19 patients: A systematic review and meta-analysis of randomized controlled trials [86]	Meta-analysis of randomized controlled trials	Multiple statins	Statin use did not affect the clinical outcomes of patients hospitalized with COVID-19.	Ia
	2022	Statin and aspirin as adjuvant therapy in hospitalised patients with SARS-CoV-2 infection: A randomised clinical trial (RESIST trial) [82]	Randomized controlled trial	Atorvastatin 40 mg daily	The use of aspirin, atorvastatin, or both in hospitalized patients with mild to moderate COVID-19 infection did not prevent clinical deterioration.	Ib
	2022	Atorvastatin versus placebo in patients with COVID-19 in intensive care: Randomized controlled trial [83]	Randomized controlled trial	Atorvastatin 20 mg daily	Atorvastatin did not affect the rate of thrombosis, treatment with extracorporeal membrane oxygenation, or mortality in patients with COVID-19 admitted to the intensive care unit.	Ib
	2022	Survival of the hospitalized patients with COVID-19 receiving atorvastatin: A randomized clinical trial [84]	Randomized controlled trial	Atorvastatin 20 mg daily	Adding atorvastatin to the standard therapy for COVID-19 worsened the clinical outcomes of hospitalized patients.	Ib
	2020	Meta-analysis of effect of statins in patients with COVID-19 [62]	Meta-analysis	Multiple statins (Atorvastatin, rosuvastatin, simvastatin, pravastatin, fluvastatin, pitavastatin)	Statins significantly reduced hazard for fatal or severe disease (HR = 0.70; 95% CI 0.53–0.94) compared to non-use of statins in COVID-19 patients.	IIa
	2021	Protective effects of statins administration in European and North American patients infected with COVID-19: A meta-analysis [64]	Meta-analysis	Multiple statins	Statin usage in Western patients hospitalized with COVID-19 was associated with nearly 40% lower odds of progressing toward severe illness or death (odds ratio: 0.59; 95% confidence interval: 0.35–0.99).	IIa
	2021	The protective association between statins use and adverse outcomes among COVID-19 patients: A systematic review and meta-analysis [66]	Meta-analysis	Multiple statins	Patients who were administered statins after their COVID-19 diagnosis were at a lower risk of mortality (HR 0.53, 95% CI: 0.46, 0.61; OR 0.57, 95% CI: 0.43, 0.75). Among non-ICU patients, statin users were at a lower risk of mortality relative to non-statin users (HR 0.53, 95% CI: 0.46, 0.62; OR 0.64, 95% CI: 0.46, 0.88).	IIa
	2021	Improved COVID-19 ICU admission and mortality outcomes following treatment with statins: A systematic review and meta-analysis [67]	Meta-analysis	Multiple statins (lovastatin OR fluvastatin OR pravastatin OR rosuvastatin OR pitavastatin OR atorvastatin OR simvastatin OR cerivastatin OR lipitor OR lescol OR lecol AND xl OR mevacor OR altoprev OR pravachol OR crestor OR zocor OR livalo.)	There were significant reductions in ICU admission (OR = 0.78, 95% CI: 0.58–1.06; *n* = 10; I ^2^ = 58.5%) and death (OR = 0.70, 95% CI: 0.55–0.88; *n* = 21; I^2^ = 82.5%) outcomes, with no significant effect on tracheal intubation (OR = 0.79; 95% CI: 0.57–1.11; *n* = 7; I^2^= 89.0%).	IIa
	2021	The use of statins was associated with reduced COVID-19 mortality: A systematic review and meta-analysis [68]	Meta-analysis	Multiple statins	The use of statins was significantly associated with decreased mortality (odds ratio [OR] = 0.71, 95% confidence interval [CI]: 0.55–0.92) and the need for IMV (OR = 0.81, 95% CI: 0.69–0.95) but was not linked to the need for ICU care (OR = 0.91, 95% CI: 0.55–1.51).	IIa
	2021	In-hospital use of statins is associated with a reduced risk of mortality in coronavirus-2019 (COVID-19): Systematic review and meta-analysis [69]	Meta-analysis	Multiple statins (atorvastatin, fluvastatin, pravastatin, simvastatin, rosuvastatin)	In-hospital use of statin was associated with a reduced risk of mortality (RR 0.54, 95% CI 0.50–0.58, *p* < 0.00001; I^2^: 0%, *p* = 0.87), while pre-admission use of statin was not associated with mortality (RR 1.18, 95% CI 0.79–1.77, *p* = 0.415; I^2^: 68.6%, *p* = 0.013).	IIa
	2021	Statins and clinical outcomes with COVID-19: Meta-analyses of observational studies [63]	Meta-analysis	Multiple statins	Univariate comparisons of statin users versus non-users found no statistically significant reduction in deaths (OR: 0.97, 95% CI: 0.92–1.03) or severity (OR: 1.09, 95% CI: 0.99–1.22), but multivariable analysis found lower OR in statin users.	IIa
	2021	Prior statin use and risk of mortality and severe disease from coronavirus disease 2019: A systematic review and meta-analysis [70]	Meta-analysis	Multiple statins	Prior statin use was associated with a lower risk of mortality (pooled aRR, 0.65 [95% confidence interval 143, 0.56–0.77], I^2^ = 84.1%) and a reduced risk of severe COVID-19 (pooled aRR, 0.73 [95% CI, 0.57–0.94].	IIa
	2021	Statins reduce mortality in patients with COVID-19: An updated meta-analysis of 147,824 patients [71]	Meta-analysis	Multiple statins (atorvastatin (71%), rosuvastatin (13%), and simvastatin (13%))	Meta-analyses of the adjusted odds ratio (aOR 0.67, 95% CI 0.52–0.86; 11 studies) and adjusted hazard ratio (aHR 0.73, 95% CI 0.58–0.91; 10 studies) showed that statins were independently associated with a significant reduction in mortality.	IIa
	2021	Statin use and mortality in COVID-19 patients: Updated systematic review and meta-analysis [73]	Meta-analysis	Multiple statins (Atorvastatin, pitavastatin, simvastatin, rosuvastatin)	The reported adjusted hazard ratios for mortality in statin users versus non-users showed a pooled estimate at 0.65 (95% confidence intervals [CI] 0.53, 0.81).	IIa
	2021	Statin use is associated with a decreased risk of mortality among patients with COVID-19 [75]	Meta-analysis	Multiple statins	Statin use was associated with a significantly decreased risk of mortality among patients with COVID-19 (RR adjusted = 0.64; 95% CI: 0.57–0.72, *p* < 0.001).	IIa
	2021	Statin therapy is associated with less ICU admissions in COVID-19 patients [76]	Meta-analysis	Multiple statin	Statin users show a lower risk of ICU admission compared to non-statin users (OR: 0.84, 95% CI: 0.72–0.99, *p* = 0.004, I^2^: 39.2%). A further meta-regression, using age as moderator variable, did not reveal any significant correlation, although a positive trend between the outcome and age was observed (β = 0.006, 95% CI: −0.006 to 0.019, Z value: 0.98, *p* = 0.32).	IIa
	2021	Statin and outcomes of coronavirus disease 2019 (COVID-19): A systematic review, meta-analysis, and meta-regression [77]	Meta-analysis	Multiple statins	Statins did not improve COVID-19 outcomes (OR 1.08, 95% CI 0.86–1.35; *p* = 0.50).	IIa
	2022	Statin use and clinical outcomes in patients with COVID-19: An updated systematic review and meta-analysis [65]	Meta-analysis	Multiple statins	The use of statin was found to significantly reduce the risk of adverse outcomes (OR 0.51; 95% CI 0.41 to 0.63, *p* < 0.0005.	IIa
	2022	Statin and mortality in COVID-19: A systematic review and meta-analysis of pooled adjusted effect estimates from propensity-matched cohorts [72]	Meta-analysis	Multiple statins	In patients receiving statin in-hospital, the study showed that it was associated with lower mortality (RR 0.71 (0.54, 0.94), *p* = 0.030.	IIa
	2022	The association between the use of statins and clinical outcomes in patients with COVID-19: A systematic review and meta-analysis [74]	Meta-analysis	Multiple statins (atorvastatin, rosuvastatin, simvastatin, pravastatin, pitavastatin)	The use of statins was associated with a significantly lower risks of all-cause mortality (HR = 0.70, 95% CI 0.58–0.84, *n* = 21,127, and OR = 0.63, 95% CI 0.51–0.79, *n* = 115,097) and the composite endpoint of severe illness (OR = 0.80, 95% CI 0.73–0.88, *n* = 10,081) in patients with COVID-19, compared to non-use of statins.	IIa
	2021	Statins and SARS-CoV-2 infection: Results of a population-based prospective cohort study of 469 749 adults from 2 Canadian provinces [53]	Prospective cohort	Multiple statins	Patients younger than 75 years on statin had the same incidence and outcomes of SARS-CoV-2; however, patients older than 75 years using statins had increased hospitalizations but lower 30-day all-cause mortality.	IIb
	2021	Pharmacological predictors of morbidity and mortality in COVID-19 [30]	Retrospective cohort	Multiple statins (rosuvastatin, atorvastatin)	Statin use was associated with lower rates of critical care admission (HR, 0.35; 95% CI, 0.17–0.73; *p* = 0.006).	IIb
	2021	Association between antecedent statin use and severe disease outcomes in COVID-19: A retrospective study with propensity score matching [35]	Retrospective cohort	Multiple statins (rosuvastatin, atorvastatin, simvastatin, pravastatin, lovastatin)	Statin users had significant reduction in mortality in the PSM cohort as well (OR, 0.56; 95% CI, 0.37–0.83; *p* = 0.004).	IIb
	2021	Association between antecedent statin use and decreased mortality in hospitalized patients with COVID-19 [36]	Retrospective cohort	Multiple statins	Statin use was significantly associated with lower odds of the primary endpoint in the propensity-matched cohort (OR 0.47, 95% CI 0.36–0.62, *p* < 0.001).	IIb
	2021	Association of pre-admission statin use with reduced in-hospital mortality in COVID-19 [43]	Retrospective cohort	Multiple statins	There was a statistically significant decrease in the odds of in-hospital mortality in patients on statins before admission (OR 0.14, 95% CI 0.03–0.61, *p* = 0.008).	IIb
	2021	Beneficial effect of statins in COVID-19-related outcomes—brief report: A national population-based cohort study [44]	Retrospective cohort	Multiple statins (atorvastatin, fluvastatin, lovastatin, pitavastatin, pravastatin, rosuvastatin, and simvastatin)	There was a significant decrease in hazard ratio associated with the use of statins (hazard ratio, 0.637 [95% CI, 0.425–0.953]; *p* = 0.0283).	IIb
	2021	Prior treatment with statins is associated with improved outcomes of patients with COVID-19: Data from the SEMI-COVID-19 Registry [45]	Retrospective cohort	Multiple statins	Continuation of statin therapy was associated with lower all-cause mortality (OR 0.67, 0.54–0.83, *p* < 0.001); lower incidence of acute kidney injury (AKI) (OR 0.76, 0.6–0.97, *p* = 0.025), acute respiratory distress syndrome (ARDS) (OR 0.78, 0.69–0.89, *p* < 0.001), and sepsis (4.82% vs. 9.85%, *p* = 0.008); and less need for invasive mechanical ventilation (IMV) (5.35% vs. 8.57, *p* < 0.001) compared to patients whose statin therapy was withdrawn during hospitalization.	IIb
	2021	Association of in-hospital use of statins, aspirin, and renin-angiotensin-aldosterone inhibitors with mortality and ICU admission due to COVID-19 [46]	Retrospective cohort	Atorvastatin	Atorvastatin was associated with reduced mortality, which persisted after adjusting for age, lockdown status, and other medications (OR: 0.18. 95% CI: 0.06–0.49, *p* = 0.001).	IIb
	2021	Identification of drugs associated with reduced severity of COVID-19—a case-control study in a large population [47]	Retrospective cohort	Rosuvastatin	Rosuvastatin is associated with significantly reduced odds for COVID-19 hospitalization (OR = 0.673, 95% CI [0.596 to 0.758], *p* < 0.001).	IIb
	2021	Promising effects of atorvastatin on mortality and need for mechanical ventilation in patients with severe COVID-19: A retrospective cohort study [48]	Retrospective cohort	Atorvastatin	In-hospital use of atorvastatin was associated with decrease in mortality (HR = 0.679, *p* = 0.005) and lower need for invasive mechanical ventilation (HR = 0.602, *p* = 0.014).	IIb
	2021	Statins in patients with COVID-19: A retrospective cohort study in Iranian COVID-19 patients [56]	Retrospective cohort	Three different types of statin were used among the 75 patients on statins: atorvastatin (94.7%), rosuvastatin (2.7%), and simvastatin (2.7%)	After propensity score matching, statin use appeared to be associated with a lower risk of morbidity [HR = 0.85, 95% CI = (0.02, 3.93), *p* = 0.762] and lower risk of death [(HR = 0.76; 95% CI = (0.16, 3.72), *p* = 0.735)]; however, these associations did not reach statistical significance. Furthermore, statin use reduced the chance of being subjected to mechanical ventilation [OR = 0.96, 95% CI = (0.61–2.99), *p* = 0.942], and patients on statins showed a more normal computed tomography scan result [OR = 0.41, 95% CI = (0.07–2.33), *p* = 0.312].	IIb
	2021	Impact of prior statin use on clinical outcomes in COVID-19 patients: Data from tertiary referral hospitals during COVID-19 pandemic in Italy [60]	Retrospective cohort	Multiple statins	Statin use was associated with more severe disease (OR 1.7, 95% CI 1.067–2.71; *p* = 0.026) but not mortality.	IIb
	2022	Effect of statin therapy on SARS-CoV-2 infection-related mortality in hospitalized patients [25]	Retrospective cohort	Categorized as high intensity (80 mg/day atorvastatin and 20 mg/day rosuvastatin) or low-moderate intensity	A lower SARS-CoV-2 infection-related mortality was observed in patients treated with ST prior to hospitalization (19.8% vs. 25.4%, χ^2^ with Yates continuity correction: *p* = 0.027).	IIb
	2022	Effects of statins on outcomes in Hispanic patients with COVID-19 [27]	Retrospective cohort	Multiple statins (rosuvastatin-atorvastatin-simvastatin)	In patients who had myocardial infarction and stroke with COVID-19, association was found between statin use and a reduced risk of mortality (aRR = 0.61, *p* = 0.005), mechanical ventilation (aRR = 0.53, *p* = 0.012), and ICU transfers (aRR = 0.81, *p* = 0.005).	IIb
	2022	The effect of statins on clinical outcome among hospitalized patients with COVID-19: A multi-centric cohort study [28]	Retrospective cohort	Multiple statins (rosuvastatin-atorvastatin-simvastatin)	Statin use was associated with lower odds of mortality in the propensity-matched cohort (OR 0.52, 95% CI 0.33–0.64, *p* < 0.001).	IIb
	2022	Association of statins and 28-day mortality rates in patients hospitalized with severe acute respiratory syndrome coronavirus 2 infection [33]	Retrospective cohort	Multiple statins (rosuvastatin-atorvastatin)	Statin use during hospitalization for SARS-CoV-2 infection was associated with reduced 28-day mortality rates (HR, 0.566; *p* = 0.008).	IIb
	2022	Statins and risk of thrombosis in critically ill patients with COVID-19: A multicenter cohort study [41]	Retrospective cohort	Multiple statins (atorvastatin (81.8%), rosuvastatin (16%)	Patients who received statin therapy had lower 30-day (HR 0.72 (95% CI 0.54, 0.97), *p* = 0.03) and in-hospital mortality (HR 0.67 (95% CI 0.51, 0.89), *p* = 0.007). Other secondary outcomes were not statistically significant between the two groups except for D-dimer levels (peak) during ICU stay.	IIb
	2022	The impact of HMG-CoA reductase inhibitors use on the clinical outcomes in critically ill patients with COVID-19: A multicenter, cohort study [42]	Retrospective cohort	Multiple statins (atorvastatin (81.3%), rosuvastatin (14.1%)	The in-hospital mortality [hazard ratio 0.69 (95% CI 0.54, 0.89), *p* = 0.004] and 30-day mortality [hazard ratio 0.75 (95% CI 0.58, 0.98), *p* = 0.03] were significantly lower in patients who received statin therapy and had lower odds of hospital-acquired pneumonia [OR 0.48 (95% CI 0.32, 0.69), *p* < 0.001].	IIb
	2022	Associations of statin use with 30-day adverse outcomes among 4 801 406 US Veterans with and without SARS-CoV-2: An observational cohort study [55]	Retrospective cohort	Multiple statins	Statin use was associated with lower odds of death at 30 days (OR 0.81 (95% CI 0.77 to 0.85)) but not with hospitalization or ICU admission. Associations were similar comparing use of each specific statin to no statin. Compared with low–moderate-intensity statin use, high-intensity statin use was not associated with lower odds of ICU admission or death.	IIb
	2022	A historical cohort study to investigation of statins safety in COVID-19 hospitalized patients [57]	Retrospective cohort	Multiple statins with most common atorvastatin and rosuvastatin.	Following adjustment of odds ratio based on multiple variables (age, sex, diabetes, hypertension status, stroke, dyslipidemia, cardiovascular diseases, chronic kidney disease (CKD), corticosteroids, renin-angiotensin-aldosterone axis inhibitors and proton pump inhibitors (PPIs)), it was shown that statins did not change mortality (95% CI, OR 0.71 (0.41–1.22), *p* = 0.22), ICU admission (95% CI, OR 1.05 (0.66–1.66), *p* = 0.835), or length of hospitalization (95% CI, OR 1.30 (0.78–2.17), *p* = 0.311).	IIb
	2022	Association between statin use and outcomes in patients with coronavirus disease 2019 (COVID-19): A nationwide cohort study [58]	Retrospective cohort	Simvastatin, atorvastatin, rosuvastatin	After adjustment for age, sex, ethnicity, socioeconomic status, and comorbidities, statin exposure was not associated with a significantly different risk of mortality (HR 0.96 (95% CI 0.78 to 1.18); severe COVID-19 infection (HR 1.16 (95% CI 0.95 to 1.41); or the composite outcome of all-cause mortality or severe COVID-19 infection (HR 1.05 (95% CI 0.89 to 1.23).	IIb
	2023	Statin therapy may protect against acute kidney injury in patients hospitalized for interstitial SARS-CoV2 pneumonia [54]	Prospective cohort	Multiple statins	Statin use did not affect critical care admission and mortality but significantly lowered the risk of developing acute kidney injury (OR 0.47, 95% CI 0.23–0.95; *p* = 0.036) and C-reactive protein (CRP) levels (*p* = 0.048).	IIb
	2023	Association of statin use with outcomes of patients admitted with COVID-19: An analysis of electronic health records using superlearner [49]	Retrospective cohort	Multiple statins	Statin users still had lower rates of the composite outcome (adjusted risk difference: −3.4%; 95% CI: −4.6% to −2.1%), ICU admissions (−3.3%; −4.5% to −2.1%), and intubation (−1.9%; −2.8% to −1.0%) but comparable inpatient deaths (0.6%; −1.3% to 0.1%).	IIb
	2023	Survival impact of previous statin therapy in patients hospitalized with COVID-19 [31]	Retrospective cohort	Multiple statins (rosuvastatin, atorvastatin, simvastatin, pravastatin, pitavastatin, fluvastatin- lovastatin).	Significant association between previous treatment with statins and lower mortality in hospitalized patients with COVID-19 (HR: 0.76; 95% CI: 0.59–0.97).	IIb
	2023	The impact of statin therapy on in-hospital prognosis and endothelial function of patients at high-to-very high cardiovascular risk admitted for COVID-19 [38]	Retrospective cohort	Multiple statins	Preadmission statin therapy was independently associated with a 75% risk reduction of intensive care unit admission/in-hospital death (adjusted hazard ratio 0.252, 95% confidence interval 0.122–0.521, *p* < 0.001).	IIb
	2023	Role of statins in clinical evolution of octogenarian patients admitted due to COVID-19 [39]	Retrospective cohort	Multiple statins (rosuvastatin, atorvastatin, pravastatin)	Pre-admission treatment with statins was associated with lower in-hospital mortality (RR 0.58 95% CI [0.41–0.83]; *p* = 0.003).	IIb
	2023	Routine statins use is associated with less adverse outcome in patients above 70 years of age admitted to hospital with COVID-19 [40]	Retrospective cohort	Multiple statins	After adjustment for potential confounders, prior statin use was associated with decreased risk for an adverse outcome (odds ratio = 0.4, 95% confidence interval 0.18–0.92, *p* = 0.03).	IIb
	2020	In-hospital use of statins is associated with a reduced risk of mortality among individuals with COVID-19 [26]	Retrospective cohort	Multiple statins (atorvastatin—83.2%, rosuvastatin 15.6%)	Based on a mixed-effect Cox model after propensity score-matching, we found that the risk for 28-day all-cause mortality was 5.2% and 9.4% in the matched statin and non-statin groups, respectively, with an adjusted hazard ratio of 0.58.	III
	2020	Relation of statin use prior to admission to severity and recovery among COVID-19 inpatients [34]	Retrospective cohort	Multiple statins	Statin use prior to admission was associated with reduced risk of severe COVID-19 (adjusted OR 0.29, 95% CI 0.11 to 0.71, *p* < 0.01) and faster time to recovery among those without severe disease (adjusted HR for recovery 2.69, 95% CI 1.36 to 5.33, *p* < 0.01).	III
	2020	Statin use is associated with lower disease severity in COVID-19 infection [37]	Retrospective cohort	Multiple statins	Logistic treatment models showed a lower chance of ICU admission for statin users when compared to non-statin users (ATET: Coeff (risk difference): −0.12 (−0.23, −0.01); *p* = 0.028).	III
	2020	Risk Factors associated with in-hospital mortality in a US national sample of patients with COVID-19 [50]	Retrospective cohort	Multiple statins	Receipt of statins (OR, 0.60; 95% CI, 0.56–0.65; *p* < 0.001) was associated with decreased odds of death.	III
	2020	Protective role of statins in COVID 19 patients: Importance of pharmacokinetic characteristics rather than intensity of action [79]	Retrospective cohort	Multiple statins (18/42 on high-intensity statin, 24/42 on low or moderate-intensity statin)	Simvastatin and atorvastatin reduced mortality in COVID-19 patients, unlike pravastatin and rosuvastatin, which did not.	III
	2021	Predictors of hospital discharge and mortality in patients with diabetes and COVID-19: Updated results from the nationwide CORONADO study [59]	Observational study	Multiple statins	In patients with diabetes hospitalized for COVID-19, statin use was associated with a higher risk of death (OR 1.42, 95% CI 1.00–2.02).	III
	2021	Association of lipid-lowering drugs with COVID-19 outcomes from a Mendelian randomization study [51]	Mendelian randomization study	Multiple statins	Higher expressions of HMG-CoA reductase (HMGCR) and HMGCR-mediated LDL cholesterol were associated with a higher risk of COVID-19 hospitalization (OR 1.38, 95% CI 1.06–1.81 and OR 1.32, 95% CI 1.00–1.74, respectively).	III
	2021	The role of lovastatin in the attenuation of COVID-19 [24]	Retrospective cohort	Lovastatin	There is a potential use of lovastatin to mitigate the inflammatory response induced by SARS-CoV-2 infection.	III
	2021	Decreased mortality rate among COVID-19 patients prescribed statins: Data from electronic health records in the US [32]	Retrospective cohort	Multiple statins (atorvastatin (Lipitor), cerivastatin (Baycol), fluvastatin (Lescol), lovastatin (Mevacor), pitavastatin (Zypitamag, Livalo or Nikita), pravastatin (Pravachol), rosuvastatin (Ezallor or Crestor), simvastatin (FloLipid or Zocor)	Statins do not increase COVID-19-related mortality and may, in fact, have a mitigating effect on the severity of the disease reflected in a slight reduction in mortality.	III
	2022	Critical influenza and COVID-19—a comparative nationwide case-control study [29]	Retrospective cohort	Multiple statins (rosuvastatin, atorvastatin, simvastatin)	Premorbid use of statin medication was associated with a reduced risk of ICU admission for both diseases.	III
	2022	The association of statins use with survival of patients with COVID-19 [52]	Retrospective cohort	Multiple statins	Continuous statin use was associated with lower in-hospital mortality compared to no statin use and discontinuation of statins.	III
	2020	Statins and SARS-CoV-2 disease: Current concepts and possible benefits [23]	Narrative review	Multiple statins (rosuvastatin- atorvastatin- simvastatin)	Early international retrospective observations suggested a beneficial effect of statin use before and during hospitalization on the clinical outcomes of COVID-19, notably critical care admission and mortality.	IV
	2021	COVID-19 and lipids. The role of lipid disorders and statin use in the prognosis of patients with SARS-CoV-2 infection [61]	Narrative review	Multiple statins	Numerous observational studies have shown potential beneficial effects of lipid-lowering treatment on the course of COVID-19 with significant improved prognosis and reduced mortality.	IV
	2021	Investigating lipid-modulating agents for prevention or treatment of COVID-19: JACC State-of-the-Art Review [81]	Narrative review	Multiple statins	Statins’ impact on COVID-19 outcomes was also studied through randomized controlled clinical trials (RCT), reportedly seventeen in 2021.	IV

Studies in humans also derived conflicting data [92]. An analysis of over 10 influenza seasons (1996 to 2006) in Canada attributed a minimal statistically significant protective effect of statin against influenza morbidity to confounding variables [93]. A retrospective study exploring the 2009 influenza pandemic in the United Kingdom did not find a statistically significant association between pre-admission statin use and severity of disease outcome [94]. An analysis of the data from the Centers for Disease Control and Prevention’s (CDC) Emerging Infections Program (EIP) failed to show a protective effect on mortality from influenza in hospitalized patients during the 2009 pandemic and, therefore, did not recommend the use of statins as an adjunct therapy for preventing death in these patients [95]. Although data from CDC EIP had previously shown reduced mortality in patients hospitalized with influenza during the season of 2007–2008 [96], they were later considered biased by unmeasured confounders [95]. Studies published since then, including those exploring large cohorts, also concluded an absence of benefits when using statins in patients with influenza [97,98] or attributed this potentially beneficial effect to a health user bias [99]. On the other hand, in a nationwide Swedish retrospective study published in 2022, the premorbid use of statin medication was associated with a reduced risk of intensive care unit (ICU) admission with an odds ratio (OR) of 0.74 (95% confidence interval (CI) 0.62–0.89; *p*-value 0.002) [29]. A 2022 meta-analysis evaluated the impact of statins on influenza prevalence among both vaccinated and unvaccinated individuals across multiple observational studies. The analysis, which included 14,997 participants, found that regular statin use was associated with a significant reduction in overall influenza prevalence (OR 0.85, 95% CI 0.73–0.99, *p* = 0.04). However, subgroup analyses revealed differential effects depending on the viral subtype—statin use was linked to a lower prevalence of H1N1 but a higher prevalence of H3N2, suggesting potential subtype-specific variations. No significant association was observed for influenza B infections. The meta-analysis reported no evidence of publication bias based on Egger’s (*p* = 0.018) and Begg’s (*p* = 0.071) tests. Regarding mortality outcomes, the same meta-analysis included data from 83,793 influenza-infected patients across six studies. Statin therapy was associated with a significant reduction in both 30-day (OR 0.61, 95% CI 0.47–0.80, *p* < 0.001) and 90-day mortality (OR 0.74, 95% CI 0.55–1.00, *p* = 0.042). The pooled analysis further confirmed a survival benefit for statin users (OR 0.68, 95% CI 0.56–0.82, *p* < 0.01), though some heterogeneity was present (I^2^ = 11.5%). Notably, Egger’s test suggested possible publication bias (*p* < 0.01) [100]. Another meta-analysis, also published in 2022, examined statin therapy’s impact on influenza-related mortality across ten observational cohort studies, including 2,390,730 patients. This analysis similarly demonstrated a significant reduction in mortality among statin users (OR 0.66, 95% CI 0.51–0.85, *p* < 0.01). While study designs varied, with some adjusting for confounders using propensity score matching, no significant publication bias was detected (Egger’s test *p* = 0.164, Begg’s test *p* = 0.421). However, high statistical heterogeneity (I^2^ = 69%) was noted, likely reflecting differences in study populations, statin dosages, and influenza seasons [101].

In summary, while some observational studies support the association of statins with improved influenza outcomes, there is still no solid clinical evidence of such benefits. Thus, future clinical trials, including hospitalized statin-naïve patients with influenza, are warranted while taking into consideration the metabolic and immunity status of recruited participants [102]. All studies are summarized in Table 2 and Table 3.

**Table 2:** Animal studies investigating the role of statins in viruses.

Pathogen	Year	Title of Study	Type of Study	Type of Statin	Key Findings Pertaining to Statins
SARS-CoV-2	2023	Target-agnostic drug prediction integrated with medical record analysis uncovers differential associations of statins with increased survival in COVID-19 patients [80]	Cell culture	Simvastatin	Simvastatin was a potent direct inhibitor of SARS-CoV-2 infected cells in vitro, unlike most other statins being less effective.
					
Influenza	2009	Evaluation of the efficacy and safety of a statin/caffeine combination against H5N1, H3N2, and H1N1 virus infection in BALB/c mice [87]	Animal study	Statin 50 μg/caffeine 200 μg	The combination of statins and caffeine was first proven to decrease lung damage and viral replication as effectively as oseltamivir and ribavirin, notably when administered preventatively.
	2012	The effect of rosuvastatin in a murine model of influenza A infection [88]	Animal study	Rosuvastatin	Statins did not affect the clearance of the influenza A virus from the lung and did not decrease the severity of the induced lung injury or related mortality.
	2012	Effect of statin treatments on highly pathogenic avian influenza H5N1, seasonal, and H1N1pdm09 virus infections in BALB/c mice [103]	Animal study	Multiple statins (simvastatin, lovastatin, mevastatin, pitavastatin, atorvastatin, or rosuvastatin) at various concentrations	The statins administered intraperitoneally or orally at any dose did not significantly enhance the total survivors relative to untreated controls.
	2013	Simvastatin treatment showed no prophylactic effect in influenza virus-infected mice [90]	Animal study	Simvastatin	Results showed that simvastatin failed to protect mice against influenza virus infection.
	2013	Simvastatin and oseltamivir combination therapy does not improve the effectiveness of oseltamivir alone following highly pathogenic avian H5N1 influenza virus infection in mice [89]	Animal study	Simvastatin	In mice infected with influenza, simvastatin did not decrease the morbidity, mortality, or viral titers and its addition to oseltamivir was no more efficient than oseltamivir alone.
	2014	Protective effect of fluvastatin on influenza virus infection [91]	Cell culture	Fluvastatin	Fluvastatin had an anti-inflammatory activity that minimally inhibited influenza virus infection.
					
Yellow fever	2021	Inhibition of orbivirus replication by fluvastatin and identification of the key elements of the mevalonate pathway involved [104]	Cell culture and animal study	Fluvastatin	Fluvastatin reduces replication of orbiviruses (bluetongue virus (BTV) and Great Island virus (GIV)) in cell culture. Pre-treatment of IFNAR(-/-) mice with fluvastatin promoted their survival upon challenge with live BTV, although only limited protection was observed.
Dengue	2009	Cholesterol biosynthesis modulation regulates dengue viral replication [105]	Cell culture	Lovastatin	Lovastatin could inhibit DEN-2 New Guinea C live virus replication in human peripheral blood mononuclear cells.
	2014	Lovastatin delays infection and increases survival rates in AG129 mice infected with dengue virus serotype 2 [106]	Animal study	Lovastatin	Mice pre-treated with lovastatin had lower dengue 2 viremia levels and increased survival. Effect was dose dependent.
	2019	Lipophilic statins inhibit Zika virus production in Vero cells [16]	Cell culture	Lipophilic statins (atorvastatin, cerivastatin, fluvastatin, lovastatin, mevastatin, and simvastatin)	Lipophilic statins (atorvastatin, cerivastatin, fluvastatin, lovastatin, mevastatin, and simvastatin) could reduce ZIKV production in vitro and result in smaller foci of infection. Early treatment with statins is more beneficial than late treatment; however, statins could not completely inhibit the entry stage of ZIKV infection. Fluvastatin was the most efficient at low concentrations in terms of anti-ZIKV capacity.
	2022	Effects of statin combinations on Zika virus infection in Vero cells [107]	Cell culture	Multiple statins	In a previous study, they found that lipophilic statins can inhibit ZIKV production in Vero cells; therefore, they looked at combinations. They found that certain combinations of atorvastatin or fluvastatin with simvastation or mevastatin may be synergistic.
	2023	Cholesterol-lowering drugs as potential antivirals: A repurposing approach against flavivirus Infections [108]	Cell culture and animal study	Atorvastatin	The combination of atorvastatin and ezetimibe had a synergistic effect against dengue 2, an additive effect against dengue 4 and Zika, and an antagonistic effect against yellow fever virus. In mice infected with dengue 2, monotherapy with atorvastatin or ezetimibe had significantly increased survival but not with the combination of both drugs.
					
Norovirus	2009	Role of cholesterol pathways in norovirus replication [109]	Cell culture	Simvastatin and lovastatin	Statins enhance LDL receptor expression, aiding cholesterol uptake in cells, potentially increasing norovirus susceptibility. Unlike statins, zaragozic acid and 6-fluoromevalonate, which block cholesterol biosynthesis via different pathways, did not affect virus replication. This raises concerns about statins’ role in norovirus infections and merits further study.
	2012	The effects of simvastatin or interferon-α on infectivity of human norovirus using a gnotobiotic pig model for the study of antivirals [110]	Animal study	Simvastatin	Simvastatin treatment elevated HuNoV infectivity in Gn pig model, likely by suppressing innate immunity and reducing cholesterol levels, aligning with previous reports. These results offer insight into the heightened HuNoV disease observed in statin-treated individuals.
	2013	Median infectious dose of human norovirus GII.4 in gnotobiotic pigs is decreased by simvastatin treatment and increased by age [111]	Animal study	Simvastatin	Simvastatin increased susceptibility to infection and more importantly the incidence of diarrhoea in Gn pigs inoculated with a GII.4 NoV variant.
	2021	Simvastatin reduces protection and intestinal T-cell responses induced by a norovirus P particle vaccine in gnotobiotic pigs [112]	Animal study	Simvastatin	In a pig model of human NoV infection, simvastatin, a cholesterol-lowering agent, was observed to impede T-cell immunity and annul the protective efficacy of P particles, shedding light on the potential impact of statins on NoV pathogenesis.
					
Hepatitis B and C	2012	Atorvastatin inhibits proliferation and apoptosis but induces senescence in hepatic myofibroblasts and thereby attenuates hepatic fibrosis in rats [113]	Cell culture	Atorvastatin	Atorvastatin inhibited the activation of hepatic stellate cells to myofibroblasts (MFB) and decreased cytokine and collagen production in MFB in vitro, thereby reduced MFB turnover and fibrogenesis.
	2013	The transcription factor KLF2 mediates hepatic endothelial protection and paracrine endothelial-stellate cell deactivation induced by statins [114]	Cell culture	Four different statins (atorvastatin, mevastatin, simvastatin, and lovastatin)	Upregulation of hepatic endothelial KLF2-derived transcriptional programs by statins confers vasoprotection and stellate cells deactivation, reinforcing the therapeutic potential of these drugs for liver diseases that course with endothelial dysfunction.
					
Measles	2009	Impaired cholesterol biosynthesis in a neuronal cell line persistently infected with measles virus [115]	Cell culture	Simvastatin	Simvastatin leads to reduction in number of giant cells and virus plaques; however, it was dose-dependent. This suggested that cholesterol synthesis and raft integrity are important factors for successful budding and production of infectious MV progeny.

### 2.3. Select Travel-Related Flavivirus Concerns: Yellow Fever, Dengue, Zika, Tick-Borne Encephalitis

Several studies have explored the potential impact of statins on flavivirus infections, including dengue virus (DENV), Zika virus (ZIKV), and yellow fever virus (YFV); however, definitive conclusions remain elusive [104,108] (Table 2 and Table 4). While preclinical research suggests that statins may influence viral replication, clinical evidence is still lacking [108]. A recent clinical trial (NCT03116802) investigated how statins affect innate immunity, as well as B-cell and T-cell responses, following live attenuated yellow fever vaccination. Although the study has been completed, its findings have yet to be published, leaving the clinical significance of statin use in flavivirus infections uncertain.

Preclinical studies have indicated potential benefits of statins in dengue virus (DENV) infection. Lovastatin has been shown to improve survival in DENV-infected mice and inhibit DENV-2 replication in human peripheral blood mononuclear cells [105,106]. However, these findings have not translated into clinical benefits. A randomized controlled trial in Vietnam (NCT01096576) assessed the safety and efficacy of lovastatin (80 mg/day for five days) in 300 adult dengue patients compared to placebo. Although the drug was well tolerated, with no significant difference in adverse events (64% vs. 55%, *p* = 0.13), it failed to improve disease severity, reduce viremia, or enhance clinical outcomes [116]. Similarly, a retrospective cohort study from Tan Tock Seng Hospital in Singapore analyzed 257 dengue patients with hyperlipidemia. Statin use did not significantly lower the risk of dengue hemorrhagic fever (DHF), dengue shock syndrome (DSS), or severe dengue (SD), suggesting no protective effect in clinical settings [117].

Research on statins against Zika virus (ZIKV) has been limited to in vitro studies. Españo et al. reported that early treatment with lipophilic statins reduces viral production, with fluvastatin demonstrating the highest efficacy [16]. Further findings from the same group suggested that combining atorvastatin or fluvastatin with mevastatin or simvastatin may enhance antiviral effects through a synergistic interaction [107]. Similarly, Osuna-Ramos et al. observed a dose-dependent reduction in ZIKV-infected cells treated with atorvastatin [108]. In contrast, only one study examined the impact of statins on acute and long-term clinical outcomes in tick-borne encephalitis, finding no significant association. Additionally, no differences were observed in cytokine or chemokine levels [118]. Notably, no studies have investigated statin use in Japanese encephalitis.

### 2.4. Haemorrhagic Fever Viruses

Limited data on the tropical virus Ebola found that statins have a restorative ability for endothelial dysfunction, the mechanism causing fluid and electrolyte imbalances [119] (Table 2 and Table 4). In Sierra Leone, reportedly, 100 Ebola patients were administered atorvastatin and irbesartan, and all adequately treated patients survived [120]. Unfortunately, the small observational report lacked details for validation.

### 2.5. GI Viruses: Norovirus

No published studies have reported on the effect of statins on hepatitis A, but some have reported influences of statins on norovirus, a significant cause of acute gastroenteritis globally. Investigations employing cell culture based on Norwalk virus replicon systems have discerned significant alterations in genes associated with lipid and carbohydrate metabolic pathways during norovirus replication [109]. Statins increased norovirus protein production and RNA levels, suggesting that they may promote norovirus activity [109]. In a pig model of human norovirus, simvastatin weakened T-cell immunity and reduced the effectiveness of a candidate vaccine while also increasing norovirus infectivity [111,112]. Conversely, inhibitors targeting cholesterol pathways lowered norovirus replication, underscoring the role of these pathways in norovirus infection dynamics [109]. Of note, a retrospective cohort study has associated statin use as a risk factor for norovirus disease, underscoring the necessity for further investigations to assess the ramifications of statins in norovirus infection [121].

### 2.6. Blood/Body Fluid-Transmitted Viruses: Hepatitis B, Hepatitis C

The potential impact of statins on liver-related health outcomes in patients with chronic viral hepatitis has garnered significant attention. Hepatitis B virus (HBV) and hepatitis C virus (HCV) account for almost two-thirds of the global burden of cirrhosis [134]. A population-wide cohort study of patients with chronic viral hepatitis based on the Hong Kong Hospital Authority database demonstrated that statin use was associated with various positive outcomes. This included a notable reduction in composite liver decompensation events (hazard ratio—HR: 0.55; 95% CI: 0.36–0.83; *p* = 0.005), ascites (HR: 0.57; 95% CI: 0.36–0.92; *p* = 0.02), and a dose-dependent decrease in mortality (HR: 0.87; 95% CI: 0.76–0.99; *p* = 0.035) relative to non-users [135]. In a meta-analysis assessing the impact of statins on liver-related health outcomes in patients with chronic viral hepatitis, the overall analysis did not find any association between mortality reduction and long-term statin treatment. However, the mortality risk was significantly reduced by 39% in statin users who were followed for over three years. Moreover, the risk of hepatocellular carcinoma (HCC), fibrosis, and cirrhosis in those on statins decreased by 53%, 45%, and 41%, respectively [125]. Another meta-analysis, including 123,445 patients, derived similar findings, where statin use was associated with a significantly reduced risk of virus-related cirrhosis and decompensation [136].

Several possible mechanisms have been explored to understand these effects [136]. Statins are speculated to possess anti-viral effects by blocking cholesterol synthesis and HBV or HCV replication [113,137]. They can downregulate the expression of pro-fibrotic cytokines while upregulating transcription factors that exert vasoprotective effects in the liver, thus reducing fibrosis and preventing progression or cirrhosis development [113,136,138]. Statins are also described to improve liver micro-circulation via upregulating Kruppel-like factor 2 expression, as well as facilitating the function of endothelial nitric oxide synthase [114,122].

Several studies have specifically investigated the relationship between statin use and the risk of HCC in patients with HBV or HCV. A meta-analysis of 10 studies revealed that statin users had a significantly reduced risk of HCC (relative risk = 0.47, 95% CI = 0.38–0.56), although substantial heterogeneity was observed [129]. When focusing on viral hepatitis studies, statin use was associated with a 53% reduction in HCC incidence (relative risk = 0.47, 95% CI = 0.43–0.50) with significant heterogeneity [129]. In hepatitis B virus- or hepatitis C virus-related cirrhotic patients, statin users exhibited a significant 55% reduction in HCC incidence (relative risk = 0.45, 95% CI = 0.30–0.61) with no heterogeneity, suggesting a more pronounced chemoprotective association in HBV- or HCV-related cirrhotic patients [129]. These findings suggest that statins may offer clinical benefits if used as chemoprophylactic agents to reduce liver cancer incidence [130].

Importantly, studies have demonstrated favorable long-term clinical outcomes of statins on chronic HBV infections [126,128]. A longitudinal study in chronic HBV patients with HBsAg seroclearance found that statin use was associated with a lower risk of cirrhosis and HCC (adjusted hazard ratio [aHR]: 0.44; 95% CI 0.20–0.96), and the risk decreased with each additional year of statin use (aHR: 0.85; 95% CI 0.75–0.97) [128]. Statin users experienced no hepatic decompensation or liver-related death/transplantation, in contrast to statin non-users, who had a proportion of such cases. Statin use was also linked to a lower risk of all-cause mortality (aHR: 0.21; 95% CI 0.08–0.53), and propensity score matching further supported the beneficial effects of statins on these outcomes [128]. A large cohort study of patients with chronic HBV found statin use (≥28 cumulative defined daily doses) to be associated with a significant reduction in the cumulative incidence of cirrhosis and decompensated cirrhosis [126]. This protective effect was dose-dependent, with greater reductions observed in patients with higher doses of statins.

Finally, a study encompassing various etiologies of cirrhosis, including HBV and HCV infection, as well as alcohol-related cirrhosis, revealed that statin use was linked to a dose-dependent decrease in the risk of decompensation, mortality, and HCC [127]. Other meta-analyses focusing on the use of statin in cirrhosis have shown similar protective effects in patients with cirrhosis on the development of HCC, decompensated cirrhosis, and liver cirrhosis progression [123,124]. These findings collectively suggest that statin therapy may offer protective benefits against cirrhosis and its complications in patients with chronic viral hepatitis and may extend to cirrhosis due to other etiologies [129,130,131,132].

### 2.7. Other Viruses: Measles and Herpesvirus

The importation of measles through travel-related transmission is of high public health concern. A study of measles virus infection in murine neuroblastoma cells found that pharmacological inhibition of cholesterol synthesis hindered viral budding during acute infection, indicating a link between cholesterol metabolism and viral replication [115]. Previous research has suggested a possible connection between cholesterol levels, statin use, and the risk of herpes zoster, with evidence indicating that cholesterol may play a role in varicella-zoster virus reactivation and spread [131,133]. Despite these data, a population-based retrospective cohort study conducted over 13 years identified that individuals using statins had a slight but significantly higher risk of herpes zoster compared to non-users (13.25 vs. 11.71 per 1000 person-years; HR: 1.13; 95% CI, 1.10–1.17). This association persisted even within a subgroup of patients with diabetes. These findings suggest a slight but notable increased risk of herpes zoster among older patients treated with statins, emphasizing the need for cautious consideration when prescribing these widely used lipid-lowering drugs, particularly in specific patient populations [132]. Furthermore, a meta-analysis of observational studies, including over two million participants, indicates that statin use may indeed elevate the risk of herpes zoster infection, with an OR of 1.18 [139]. Finally, we found no studies on the effect of statins on rabies virus, but in vitro studies have demonstrated that statins inhibit poliovirus replication through multiple pathways, thereby reducing infection [104,140].

## 3. Limitations

One crucial aspect to consider when evaluating the use of statins in viral infections is the potential for confounding variables introduced by the underlying comorbidities of individuals prescribed statins. Many patients prescribed statins have a history of coronary artery disease (CAD), metabolic syndrome, or a prior stroke. These conditions can significantly impact the severity and can be risk factors for infections impacting their clinical outcomes. For instance, CAD is associated with systemic inflammation, which may influence immune responses during a viral infection [141]. Metabolic syndrome, characterized by obesity, insulin resistance, and dyslipidemia, can affect the host’s immune function, potentially confounding the observed effects of statins [142]. Moreover, patients with viral infections also face an elevated risk of stroke, which can further influence decisions regarding statin therapy [143,144]. Age is another key factor, as older adults—who are more likely to be prescribed statins—also have a heightened risk of severe viral infections due to immune system decline and increased systemic inflammation. These overlapping factors make it challenging to distinguish the direct effects of statins from the overall health status of the patient population [61,78]. Therefore, when evaluating the role of statins in viral infections, it is crucial to account for these comorbidities, as they may impact the observed benefits or risks, complicating data interpretation. Additionally, differences in statin type, dosage, and duration across studies further hinder direct comparisons and limit the ability to draw definitive conclusions.

Further research is necessary to better understand how statins interact with comorbidities in the context of viral infections. RCTs are essential to establish causality, determine optimal dosing strategies, and identify patient populations that may benefit most. Given the conflicting evidence regarding statins and viral infection outcomes, well-designed prospective studies should evaluate their role beyond lipid-lowering, particularly in modulating inflammatory pathways, cytokine responses, and endothelial function in diseases such as influenza, dengue, and coronaviruses. Additionally, stratified analyses in clinical trials should assess whether specific subgroups—such as immunocompromised individuals, older adults, or those with metabolic conditions—experience differential effects from statin therapy. Our research team aimed to examine atorvastatin as an adjunctive therapy in hospitalized COVID-19 patients (NCT04380402, STATCO19) to determine whether atorvastatin can improve clinical outcomes by mitigating inflammation and endothelial dysfunction, potentially informing future treatment strategies. Further studies exploring statins in viral pneumonia, emerging infections, and their potential synergy with existing antiviral therapies are warranted.

## 4. Conclusions

The SARS-CoV-2 pandemic spread rapidly through international travel and remains a risk to travelers. Repurposing existing medications became a fruitful strategy for identifying effective treatments for the novel viral infection. The effect of statins on COVID-19 stimulated much interest because of possible immunomodulatory properties, but the studies found variable outcomes. Our assessment of statins’ influence on influenza also identified limited and variable effects of statins on influenza virus infections. Data are scarce or non-existent on other viral infections with travel-related interest, such as YF, dengue, Zika, tick-borne encephalitis, hemorrhagic fever viruses, hepatitis A, measles, Japanese encephalitis, rabies, and poliomyelitis, and limited data associated excess mortality from norovirus disease with the use of statins. Unfortunately, the non-uniformity of statins examined and the inconsistencies among study designs led to no generalizable conclusions. Nevertheless, while this review found no data on statins’ effect on acute HBV or HCV infections, accumulating data support the beneficial outcomes of statin treatment on chronic HBV and HCV. Clearly, the impact of statins on select travel-related viral infections merits more systematic prospective research to determine whether this widely used class of medications has a role in treating these infections.

## Figures and Tables

**Table 3:** Studies investigating the role of statins in influenza.

Pathogen	Year	Title of Study	Type of Study	Type of Statin	Key Findings Pertaining to Statins	Category of Evidence
Influenza	2011	Pre-admission statin use and in-hospital severity of 2009 pandemic influenza A(H1N1) disease [94]	Retrospective cohort	Multiple statins	No statistically significant association between pre-admission statin use and severity of outcome after adjustment for age and sex [adjusted OR: 0.81 (95% CI 0.46–1.38); *n* = 571].	IIb
	2012	Association between use of statins and mortality among patients hospitalized with laboratory-confirmed influenza virus infections: A multistate study [96]	Retrospective cohort	Multiple statins	In a multivariable logistic regression model, administration of statins prior to or during hospitalization was associated with protective odds of death (adjusted odds ratio, 0.59 [95% confidence interval, 0.38–0.92]) when adjusting for age; race; cardiovascular, lung, and renal disease; influenza vaccination; and antiviral administration.	IIb
	2022	The effect of statins on the prevalence and mortality of influenza virus infection: A systematic review and meta-analysis [100]	Meta-analysis	Multiple statins	In flu-vaccinated and unvaccinated subjects, the regular use of statins significantly decreased influenza prevalence (OR 0.85, 95% CI 0.73–0.99; *p* = 0.04), 30-day mortality (OR 0.61, 95% CI 0.47, 0.80; *p* < 0.001), and 90-day mortality after the diagnosis (OR 0.74, 95% CI 0.55, 1.00; *p* = 0.042).	IIb
	2022	Statins and influenza mortality: Systematic review and meta-analysis [101]	Meta-analysis	Multiple statins	Statin therapy was associated with decreased mortality (OR 0.66; 95% CI 0.51–0.85; *p* < 0.01).	IIb
	2009	Influenza morbidity and mortality in elderly patients receiving statins: A cohort study [93]	Retrospective cohort	Multiple statins: atorvastatin, cerivastatin, fluvastatin, lovastatin, pravastatin, and simvastatin	An analysis of over 10 influenza seasons (1996 to 2006) in Canada showed that the minimal statistically significant protective effect of statin against influenza morbidity was probably due to confounding variables.	III
	2015	Statin treatment and mortality: Propensity score-matched analyses of 2007–2008 and 2009–2010 laboratory-confirmed influenza hospitalizations [95]	Retrospective cohort	Data on statin dose or frequency of administration were not collected	A propensity score-matched analysis of influenza hospitalization data from two seasons suggested a protective effect of statins against mortality in 2007–2008 but not during the 2009 pandemic, with sensitivity analysis indicating potential influence from unmeasured confounders. This analysis does not support using statins as an adjunct treatment for preventing death among persons hospitalized for influenza.	III
	2017	The effect of statins on influenza-like illness morbidity and mortality [99]	Retrospective cohort	Multiple statins	The potentially beneficial effect of statins on influenza-related adverse outcomes may be explained by a healthy user bias.	III
	2018	Statin use and risks of influenza-related outcomes among older adults receiving standard-dose or high-dose influenza vaccines through Medicare during 2010–2015 [98]	Retrospective cohort	Multiple statins	Statin use around the time of vaccination did not affect the risk of influenza-related medical encounters in older adults.	III
	2019	Statins and outcomes of hospitalized patients with laboratory-confirmed 2017–2018 influenza [97]	Retrospective cohort	Multiple statins	Statin use was not associated with mortality benefit in patients with influenza.	III
	2022	Critical influenza and COVID-19—A comparative nationwide case-control study [29]	Case-control study	Multiple statins	The premorbid use of statin was associated with a reduced risk of intensive care unit (ICU) admission with an odds ratio (OR) of 0.74 (95% confidence interval (CI) 0.62–0.89; *p*-value 0.002).	III
	2013	Adjunctive therapies and immunomodulatory agents in the management of severe influenza [92]	Narrative review	Multiple statins	Epidemiologic studies on the impact of statin use in influenza patients have shown mixed results. While some studies suggest a small protective effect against pneumonia hospitalization and mortality, others found no significant association after adjusting for confounding factors.	IV

**Table 4:** Studies investigating the role of statins in other viruses.

Pathogen	Year	Title of Study	Type of Study	Type of Statin	Key Findings Pertaining to Statins	Category of Evidence
Dengue	2015	Lovastatin for the treatment of adult patients with dengue: A randomized, double-blind, placebo-controlled trial [116]	Randomized controlled trial	Lovastatin	RCT of lovastatin in patients with Dengue with the primary outcome being safety. Lovastatin was safe and well tolerated in adults with dengue. However, although the study was not powered to address efficacy, they found no evidence of a beneficial effect on any of the clinical manifestations or on dengue viremia.	Ib
	2023	Hyperlipidemia, statin use and dengue severity [117]	Retrospective cohort	Multiple statins	Compared dengue patients with hyperlipidemia on the basis of statin use. Outcomes were development of dengue hemorrhagic fever (DHF) or shock syndrome (DSS) and severe dengue (SD). A total of 257 dengue patients were included; 191 (74.3%) were statin users and 66 (25.7%) were non-users. No significant difference detected in risk of DHF/DSS (adjusted risk ratio [aRR] = 0.66, 95% confidence interval [CI]: 0.41–1.08, *p* = 0.10) or SD (aRR = 1.43, 95% CI: 0.84–2.43, *p* = 0.19).	IIb
Tick-borne encephalitis	2018	Impact of pre-existing treatment with statins on the course and outcome of tick-borne encephalitis [118]	Retrospective cohort	Multiple statins	A total of 700 adult patients with tick-borne encephalitis of whom 77 (11%) were being treated with statins, along with 410 other patients, of whom 53 (13%) were receiving statins. Multivariable analyses found no statistically significant association between statin usage and having a milder acute illness, prognosis, or long-term symptoms.	III
						
Ebola	2015	Treating the host response to Ebola virus disease with generic statins and angiotensin receptor blockers [119]	Narrative review	Atorvastatin 40 mg/day	In Sierra Leone, approximately 100 Ebola patients were treated with this combination, and reports indicate that survival was greatly improved. Unfortunately, supervising physicians and health officials in Sierra Leone have not released reports of the treatment results, although they exchanged letters and memoranda describing their experience, with one letter noting “remarkable improvement” on treatment.	IV
	2015	Treating Ebola patients: A ‘bottom up’ approach using generic statins and angiotensin receptor blockers [120]	Narrative review	Atorvastatin 40 mg/day	Atorvastatin and irbesartan reduced Ebola mortality in Sierra Leone. The treatment was also safe and restored endothelial barrier integrity.	IV
						
Norovirus	2010	Norovirus disease associated with excess mortality and use of statins: A retrospective cohort study of an outbreak following a pilgrimage to Lourdes [121]	Retrospective cohort	Multiple statins	A retrospective cohort study identified statin use as a risk factor for norovirus disease, underscoring the necessity for further investigations to assess the ramifications of statins in NoV infection.	III
						
Hepatitis B and C	2004	Simvastatin enhances hepatic nitric oxide production and decreases the hepatic vascular tone in patients with cirrhosis [122]	Randomized controlled trial	Simvastatin	Simvastatin administration increases the hepatosplanchnic output of nitric oxide products and decreases hepatic resistance in patients with cirrhosis.	Ib
	2017	Statin use and risk of cirrhosis and related complications in patients with chronic liver diseases: A systematic review and meta-analysis [123]	Meta-analysis	Multiple statins	In patients with cirrhosis, statin use was associated with 46% lower risk of hepatic decompensation (four studies; RR, 0.54; 95% CI, 0.46–0.62; I^2^ = 0%; moderate-quality evidence), and 46% lower mortality (5 studies; RR, 0.54; 95% CI, 0.47–0.61; I^2^ = 10%; moderate-quality evidence).	Ib
	2019	Comprehensive evaluation of effects and safety of statin on the progression of liver cirrhosis: A systematic review and meta-analysis [124]	Meta-analysis	Multiple statins	For a long-term follow-up, statin treatment surprisingly decreased mortality rate (HR = 0.782, 95% CI: 0.718–0.846, I^2^ > 50%) and lowered the occurrence of hepatocellular carcinoma (HR = 0.75, 95% CI: 0.64–0.86, I^2^ > 50%) in liver cirrhosis.	Ib
	2021	Statin therapy in chronic viral hepatitis: A systematic review and meta-analysis of nine studies with 195,602 participants [125]	Meta-analysis	Multiple statins	In a meta-analysis of statin effects on liver health in CVH patients, long-term statin use did not show overall mortality reduction. However, mortality risk dropped by 39% in statin users followed for over three years. Additionally, statin therapy was associated with significant reductions in hepatocellular carcinoma risk by 53%, fibrosis by 45%, and cirrhosis by 41%.	Ib
	2016	Statins reduce the risk of cirrhosis and its decompensation in chronic hepatitis B patients: A nationwide cohort study [126]	Retrospective cohort	Multiple statins	CHB patients using statins had a significantly lower cumulative incidence of cirrhosis (relative risk = 0.433; 95% confidence interval (CI) = 0.344–0.515; modified log-rank test, *p* < 0.001) and decompensated cirrhosis (relative risk = 0.468; 95% CI = 0.344–0.637; *p* < 0.001) compared with patients not using statins (after adjustment for competing mortality).	IIb
	2017	Statins decrease the risk of decompensation in hepatitis B virus- and hepatitis C virus-related cirrhosis: A population-based study [127]	Retrospective cohort	Multiple statins	Among patients with cirrhosis, statin use decreased the risk of decompensation, mortality, and HCC in a dose-dependent manner (*p* for trend <0.0001, <0.0001, and 0.009, respectively). Regression analysis revealed a lower risk of decompensation among statin users with cirrhosis due to chronic HBV (adjusted hazard ratio [HR], 0.39; 95% confidence interval [CI], 0.25–0.62) or HCV infection (HR, 0.51; 95% CI, 0.29–0.93).	IIb
	2021	Statins associate with better clinical outcomes in chronic hepatitis B patients with HBsAg seroclearance [128]	Retrospective cohort	Simvastatin, atorvastatin, and rosuvastatin	Statins were associated with lower cirrhosis/HCC risk in HbsAg seroclearance patients (adjusted hazard ratio [aHR]: 0.44; 95% CI 0.20–0.96; aHR for every 1-year increase in use: 0.85; 95% CI 0.75–0.97). Statin users had no hepatic decompensation or liver-related death/transplantation (vs 18/778 [2.3%] and 18/784 [2.3%] cases in statin non-users, respectively). Statins were also associated with lower all-cause mortality risk (aHR: 0.21; 95% CI 0.08–0.53).	IIb
	2022	Statins in hepatitis B or C patients is associated with reduced hepatocellular carcinoma risk: A systematic review and meta-analysis [129]	Meta-analysis	Multiple statins	Statin users had a significantly lower risk of hepatocellular carcinoma (relative risk = 0.47, 95% CI = 0.38–0.56) with significant heterogeneity. In seven hepatitis studies, using statin was associated with a 53% reduction in the incidence of hepatocellular carcinoma (relative risk = 0.47, 95% CI = 0.43–0.50) with substantial heterogeneity. In three cirrhosis studies, the incidence of hepatocellular carcinoma in statin users was significantly reduced by 55% (relative risk = 0.45, 95% CI = 0.30–0.61) with no heterogeneity.	IIb
	2022	Can statins lessen the burden of virus mediated cancers? [130]	Narrative review	Multiple statins	Studies of populations with HBV and HCV suggest a protective, dose-dependent effect of statins on hepatocellular carcinoma risk and support the theory that statins may offer clinical benefit if used as chemoprophylactic agents to reduce liver cancer incidence.	IV
						
Herpes simplex	2005	Statins lower the risk of developing Alzheimer’s disease by limiting lipid raft endocytosis and decreasing the neuronal spread of herpes simplex virus type 1 [131]	Narrative review	Multiple statins	Long-term statin therapy protects individuals from Alzheimer’s disease by reducing the neuronal spread of HSV-1 via lipid raft domain pathways.	IV
Varicella-Zoster	2014	Statins and the risk of herpes zoster: A population-based cohort study [132]	Retrospective cohort	Multiple statins	A population-based retrospective cohort study conducted over 13 years identified that older patients treated with statins had a small but notably increased risk of herpes zoster with a (hazard ratio) HR of 1.13. This association persisted even within a subgroup of patients with diabetes (HR—1.18)	IIb
	2009	High serum cholesterol levels are associated with herpes zoster infection after heart transplantation [133]	Case-control study	Multiple statins	A case-control analysis has indicated a link between elevated blood cholesterol levels and the occurrence of herpes zoster infection in individuals who had undergone heart transplantation. This study also found that cholesterol could trigger the reactivation and dissemination of VZV in vivo.	III

## Abbreviations

aHR	adjusted hazard ratio
APC	antigen-presenting cells
CAD	coronary artery disease
CCR-5	C-C chemokine receptor type 5
CDC	Centers for Disease Control and Prevention
CI	confidence interval
CRP	C-reactive protein
DENV	dengue virus
DHS	dengue hemorrhagic fever
DS	severe dengue
DSSL	dengue shock syndrome
EIP	Emerging Infections Program
HBV	hepatitis B Virus
HCC	hepatocellular carcinoma
HCV	hepatitis C virus
HMG-CoA	hydroxymethylglutaryl-CoA
HMG-CoAR	HMG-CoA reductase
HR	hazard ratio
ICU	intensive care unit
LDL	low-density lipoprotein
OR	odds ratio
RCT	randomized controlled trials
US	United States
YFV	yellow fever virus
ZKV	Zika virus
